# Growth pattern trajectories in boys with Duchenne muscular dystrophy

**DOI:** 10.1186/s13023-021-02158-9

**Published:** 2022-01-24

**Authors:** Georgia Stimpson, Sarah Raquq, Mary Chesshyre, Mary Fewtrell, Deborah Ridout, Anna Sarkozy, Adnan Manzur, Vandana Ayyar Gupta, Ramona De Amicis, Francesco Muntoni, Giovanni Baranello, the NorthStar Network, Gautam Ambegaonkar, Zoya Alhaswani, Alex Baxter, Anne-Marie Childs, Gabby Chow, Christian De Goede, Miguel Fernandez, Frances Gibbon, Vasantha Gowda, Michela Guglieri, Tony Hart, Gemunu Hewawitharana, Iain Horrocks, Imelda Hughes, Marjorie Illingworth, Deepa Krishnakumar, Anirban Majumdar, Chiara Marini-Bettolo, Min Ong, Deepak Parasuraman, Sithara Ramdas, Laurent Servais, Kate Skone, Stefan Spinty, Elma Stephens, Volker Straub, Sandya Tirupathi, Neil Thomas, Tracey Willis, Cathy White, Jarod Wong, Elizabeth Wraige, Kayal Vijayakumar, Karen Naismith

**Affiliations:** 1 Developmental Neuroscience Research and Teaching Department, Faculty of Population Health Sciences, Dubowitz Neuromuscular Centre, UCL Great Ormond Street Institute of Child Health, London, UK; 2 Population, Policy and Practice Department, Faculty of Population Health Sciences, UCL Great Ormond Street Institute of Child Health, London, UK; 3 Dubowitz Neuromuscular Centre, UCL Great Ormond Street Institute of Child Health and Great Ormond Street Hospital, London, UK; 4 International Centre for the Assessment of Nutritional Status (ICANS), Department of Food, Environmental and Nutritional Sciences (DeFENS), University of Milan, Milan, Italy; 5 NIHR Great Ormond Street Hospital Biomedical Research Centre, Great Ormond Street Hospital NHS Foundation Trust, UCL Great Ormond Street Institute of Child Health, 30 Guilford Street, London, WC1N 1EH UK; 6 Department of Paediatric Neurology, Royal Belfast Hospital for Sick Children, Belfast, UK; 7 University Hospitals Birmingham NHSFT, UK, Birmingham, UK; 8 Birmingham Children’s Hospital, Birmingham, UK; 9 Royal Hospital for Children, Bristol, UK; 10 Department of Child Health, Paed Neurology, University Hospital of Wales, Cardiff, UK; 11 Department of Paediatrics, Ninewells Hospital, Dundee, UK; 12 Department of Community Child Health, Royal Hospital for Sick Children, Edinburgh, UK; 13 Fraser of Allander Neurosciences Unit, Royal Hospital for Sick Children, Glasgow, UK; 14 Leeds Children Hospital, London, UK; 15 Alder Hey Children’s Hospital, Liverpool, UK; 16 Evelina Children’s Hospital, London, UK; 17 Royal Manchester Children’s Hospital, Central Manchester University Hospitals, NHS Foundation Trust, Manchester, UK; 18 John Walton Muscular Dystrophy Research Centre, Newcastle University, Newcastle upon Tyne, UK; 19 Queens Medical Centre, University Hospital, Nottingham, UK; 20 The Robert Jones and Agnes Hunt Orthopaedic Hospital, Oswestry, UK; 21 MDUK Oxford Neuromuscular Centre, Department of Paediatrics, University of Oxford, Oxford, UK; 22 Royal Preston Hospital, Preston, UK; 23 Sheffield Children’s Hospital, Sheffield, UK; 24 Department of Neurology, Southampton Children’s Hospital, Southampton, UK; 25 Morriston Hospital, Heol Maes Eglwys, Swansea, UK; 26 Department of Paediatric Neurology, Leicester Royal Infirmary, Leicester, UK; 27 Royal Aberdeen Children’s Hospital, Aberdeen, UK; 28 Department of Paediatric Neurology, Cambridge University Hospitals NHS Foundation Trust, Cambridge, UK; 29 Department of Paediatric Neurology, University Hospitals Bristol NHS Foundation Trust, Bristol, UK

**Keywords:** Duchenne muscular dystrophy, Growth, Isoforms, Glucocorticoids, Prednisolone, Deflazacort

## Abstract

**Objectives:**

The objective of this study is to analyse retrospective, observational, longitudinal growth (weight, height and BMI) data in ambulatory boys aged 5–12 years with Duchenne muscular dystrophy (DMD).

**Background:**

We considered glucocorticoids (GC) use, dystrophin isoforms and amenability to exon 8, 44, 45, 51 and 53 skipping drug subgroups, and the impact of growth on loss of ambulation. We analysed 598 boys, with 2604 observations. This analysis considered patients from the UK NorthStar database (2003–2020) on one of five regimes: “GC naïve”, “deflazacort daily” (DD), “deflazacort intermittent” (DI), “prednisolone daily” (PD) and “prednisolone intermittent” (PI). A random slope model was used to model the weight, height and BMI SD scores (using the UK90).

**Results:**

The daily regime subgroups had significant yearly height stunting compared to the GC naïve subgroup. Notably, the average height change for the DD subgroup was 0.25 SD (95% CI − 0.30, − 0.21) less than reference values. Those with affected expression of Dp427, Dp140 and Dp71 isoforms were 0.77 (95% CI 0.3, 1.24) and 0.82 (95% CI 1.28, 0.36) SD shorter than those with Dp427 and/or Dp140 expression affected respectively. Increased weight was not associated with earlier loss of ambulation, but taller boys still ambulant between the age of 10 and 11 years were more at risk of losing ambulation.

**Conclusion:**

These findings may provide further guidance to clinicians when counselling and discussing GCs commencement with patients and their carers and may represent a benchmark set of data to evaluate the effects of new generations of GC.

## Background

Duchenne muscular dystrophy (DMD) is a severe, progressive, X-linked neuromuscular disorder that mainly affects males. Depending on the site of mutations/ deletions on the dystrophin gene (DMD), the expression of different dystrophin isoforms may be variably affected, either the full-length dystrophin protein (Dp427) mainly expressed in the muscle and/or other shorter isoforms (Dp71, Dp116, Dp140, Dp260) that are expressed differentially in the body. Specifically, Dp140 is predominantly expressed in the central nervous system, while Dp70 is expressed in the brain and ubiquitously. DMD is characterised by progressive muscle wasting, weakness and consequent fibro-fatty replacement in skeletal muscle fibres [1]. There are an increasing number of treatments for specific subpopulations of DMD patients, which have been approved by the US Food and Drug Administration (FDA) or are currently in clinical trial phases. These include Eteplirsen [2, 3], Casimersen [4], Golodirsen [5], Viltolarsen [6] and Ataluren [7–9]. However, glucocorticoids (GC) are currently the only widely available recommended treatment in the DMD care considerations [10]. Increasing evidence suggests that GC can prolong survival and ambulation, as well as upper limb and cardiorespiratory function in the non-ambulatory stage [11–13]. However, chronic GC use is associated with side effects, including weight gain, raised blood pressure, behaviour changes, cataracts, osteoporosis, stunted growth, delayed puberty and other endocrine complications. Nevertheless, there has been a trend to start patients on GC treatment much earlier, usually between ages 4 and 6 years. This is due to the reported motor function benefits of initiating GC at a younger age [14]. GC are typically prescribed to DMD patients either as prednisone/prednisolone or deflazacort at dose of 0.75 mg/kg/d or 0.9 mg/kg/d, respectively, with the choice of a daily or intermittent regime based on individual patient characteristics and with the goal of limiting possible side effects. The optimum GC type and regimen for DMD management is debated, and results from a global, randomized, double-blinded trial of daily prednisone, daily deflazacort, and 10-days-on/10-days-off prednisone are pending (www.for-dmd.org). There are also other GC treatment regimens that have been prescribed which are less common in the UK, such as the weekend only regime [15].

Data from real-world registries and natural history studies can be valuable in guiding clinicians when prescribing GC type and regimen to DMD patients, as well as provide important benchmark information for new generations of GC with differential mechanisms of action designed to limit mineralocorticoid-related side effects [16]. The main considerations when deciding the GC treatment for a boy recently diagnosed with DMD are the possible long-term effect on weight, height and BMI, and the impact on motor function.

The aims of this study were: (1) to describe the growth pattern trajectories of boys with DMD either GC-naïve or treated with intermittent/daily prednisolone or deflazacort within the UK NorthStar Clinical Network and database, which is internationally one of the largest DMD data sets; (2) to investigate the impact of the DMD genotype on growth, including the effects of different DMD mutations amenable to skipping of exons 8, 44, 45, 51 and 53, and those affecting different dystrophin isoform expression; and (3) to explore the association between growth patterns and loss of ambulation.

## Methods

The NorthStar database is a national database, established in October 2006 to systematically collect longitudinal data from children with DMD across 23 paediatric neuromuscular centres in the UK. The diagnosis is confirmed in most cases by DNA diagnostic technique covering all DMD gene exons and/or a muscle biopsy. Pathogenic variants were classified according to the Leiden Open Variation Database [17]. Clinical and functional assessment data are collected and recorded on NorthStar specific forms by doctors and physiotherapists at six-monthly intervals. There is a regular national training programme and standard operating procedures are used at each site. The NorthStar forms are stored in an electronic database managed by CertusLtd [14, 18]. Clinical data is only collected after written informed consent has been obtained and the NorthStar project has the approval of the Caldicott Guardian. All clinical assessments are conducted according to the principles of the Declaration of Helsinki (2000) and the Principles of Good Clinical Practice. All patients considered in the analysis were treated according to the standards of care [10], including therapy with GC administered either as daily or intermittent (10 days on, 10 days off) prednisolone or deflazacort.

### Participants

**Fig. 1:**
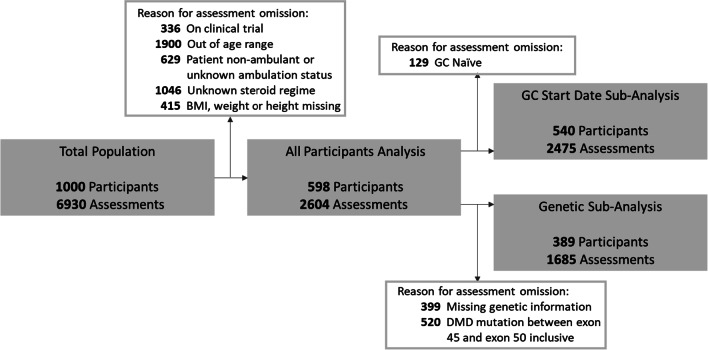
Description of data cleaning process and sample size for the main analysis and sub-analyses

From the full NorthStar population, we identified 598 DMD patients, with 2,604 assessments of height, weight and BMI records from 2006 to 2020. Participants with in-frame DMD deletions or duplications, a Becker or Intermediate phenotype and manifesting DMD female carriers were excluded. Clinical trial participants were excluded from the analysis. This cohort selection process is documented in Fig. 1. The cohort included participants aged ≥ 5 and ≤ 12 years, as this is the typical window where patients are on GCs and ambulatory. In addition, included participants were ambulant and fell into one of the following majority GC treatment groups: “GC naïve”, “deflazacort daily”, “deflazacort intermittent”, “prednisolone daily” and “prednisolone intermittent”. The mean age of GC initiation was 5.8 years (standard deviation of 1.5), which was consistent between the different treatment groups, as seen in Table 2. Standing height was recorded with an appropriate stadiometre in all participants across all sites by using a standardized procedure, as defined by the Royal College of Paediatrics and Child Health (https://www.rcpch.ac.uk/resources/uk-who-growth-charts-guidance-health-professionals).

### DMD pathogenic variant

508 participants had DMD genotype details available. We used two different criteria to assess influence of genotype on DMD. Firstly, participants were grouped into 3 groups based on predicted DMD mutation effects on dystrophin isoform expression as follows: Group 1 (Dp427 absent, Dp140/Dp71 present, n = 210); group 2 (Dp427/Dp140 absent, Dp71 present, n = 157); and group 3 (Dp427/Dp140/Dp71 absent, n = 22). Participants with DMD mutations upstream of intron 44 were considered to be Dp427 negative and Dp140/Dp71 positive (group 1), patients with DMD mutations in the region from exon 51 to exon 62 inclusive, and not exon 63 or downstream of exon 63 were considered to be Dp427/Dp140 negative and Dp71 positive (group 2) and patients with DMD mutations involving exon 63 or the genomic region downstream of exon 63 were considered to be Dp427/Dp140/Dp71 negative (group 3) [19]. Patients with DMD mutations between exon 45 and exon 51 inclusive were excluded from the analysis of the associations of growth parameters with predicted dystrophin isoform involvement due to it being difficult to predict their effect on Dp140 expression as the Dp140 promoter is located in intron 44 and the translation start site for Dp140 is located in exon 51 [20]. Secondly, DMD deletions were classified according to their amenability to skipping of exons 8, 44, 45, 51, and 53.

### Loss of ambulation

Loss of ambulation time was defined as the first visit where the patients were recorded as non-ambulant, either by the clinician recording the patient as non-ambulant, using the non-ambulant assessment form or the patient scoring 0 for the NorthStar Ambulatory Assessment subitem ‘walk’ (unable to walk unassisted).

### Statistical methods

For each of the three measures of growth (weight, height and BMI), we converted the data to standardised scores (SD scores) using the British 1990 growth reference (UK90) [21]. A mixed-effects random slopes model, which accounts for repeated measurements on the same patients, was fitted to the SD score data. The GC use was summarised as the compound type (deflazacort or prednisolone) and regime (daily, intermittent or naïve) the participant was taking for the longest duration from the age of start GC up to and including 12 years. We compared growth trajectories between the five GC regimes by including an interaction term for GC regime and age, and described the estimated SD scores at age 5 years. We compared boys on prednisolone vs. deflazacort and those on daily vs. intermittent regimes. A subgroup analysis was performed including boys with genetic information available, considering the associations between predicted dystrophin isoform involvement or amenability to exon skipping on the growth measures at 5 years of age and yearly rate of change of growth. A further subgroup analysis was performed for those boys who had started GCs, looking at the effect of the age at starting GCs on the growth measures at age 5 and the yearly change in growth measures. By considering GC starting age and age, GC exposure was implicitly adjusted for. A sensitivity analysis was undertaken to investigate if results were similar in the population of patients who did not switch GC regime before the age of 12. The height and weight of ambulant boys in 6 cross-sectional age brackets (5–6, 6–7, 7–8, 8–9, 9–10, 10–11) were used in a univariate Cox Proportional Hazard model analysis to assess the association between height and weight in different age brackets and risk of loss of ambulation during follow up.

## Results

The analysis included 598 boys with DMD, with 2,604 total observations. 203 participants had observed loss of ambulation. Participants had a median of 4 visits, with the maximum number of visits being 17. The mean follow-up duration was 2.5 years. A summary of the baseline (participants first visit with height and weight recorded) cross-sectional and longitudinal data is presented in Tables 1 and 2 respectively. Most of the patients are on GC, with the majority in the daily prednisolone group. As this data is observational, these results are comments on observed growth trends in specific subpopulations only, they should not be used to describe the effect of treatment factors. The estimated mean value at age 5 years of age and mean annualised change for height, weight and for the five GC groups and the comparisons between GC regime, type and starting age is shown in Table 2. Overall, 251 participants switched either GC regime or type, with 61 switching GC type and 222 switching GC regime. The sensitivity analysis showed no significant difference in parameter estimates when only the patients who did not switch GC regime were considered.

**Table 1:** Baseline (first visit with recorded weight SD and height SD) characteristics of all patients included in the longitudinal analysis

All participants (N = 598)	Mean	Standard deviation
Age	6.6	1.5
NSAA total	22.6	6.9
Weight	23.16	6.48
Weight SD	− 0.02	1.30
Height	113.80	9.32
Height SD	− 1.12	1.14
BMI	17.61	2.71
BMI SD	0.97	1.18

**Table 2:** Summary of patient-level characteristics

	All participants (N = 598, M = 2604)
Age (years, mean ± sd)	8.0 ± 1.8
Age range	5–12.0
Duration of follow-up (years, mean ± sd)	2.5 ± 1.9
GC (n (%))	
GC naïve	58 (10%)
GC started	540 (90%)
Prednisolone	373 (62%)
Daily	202 (34%)
Intermittent	171 (29%)
Deflazacort	167 (28%)
Daily	121 (20%)
Intermittent	46 (8%)
Deletion group (n (%))	
Group 1	210 (35%)
Group 2	157 (26%)
Group 3	22 (4%)
Exon skipping amenable (n (%))	
Skip 8	12 (2%)
Skip 44	47 (8%)
Skip 45	60 (10%)
Skip 51	68 (11%)
Skip 53	46 (8%)
NSAA score (mean ± sd)	21.7 ± 7.7
NSAA range	0–34
GC start age (mean ± sd)	5.8 ± 1.5
Prednisolone Daily	5.8 ± 1.5
Prednisolone Intermittent	5.9 ± 1.5
Deflazacort Daily	5.6 ± 1.5
Deflazacort Intermittent	6.0 ± 1.6

### Weight

**Fig. 2:**
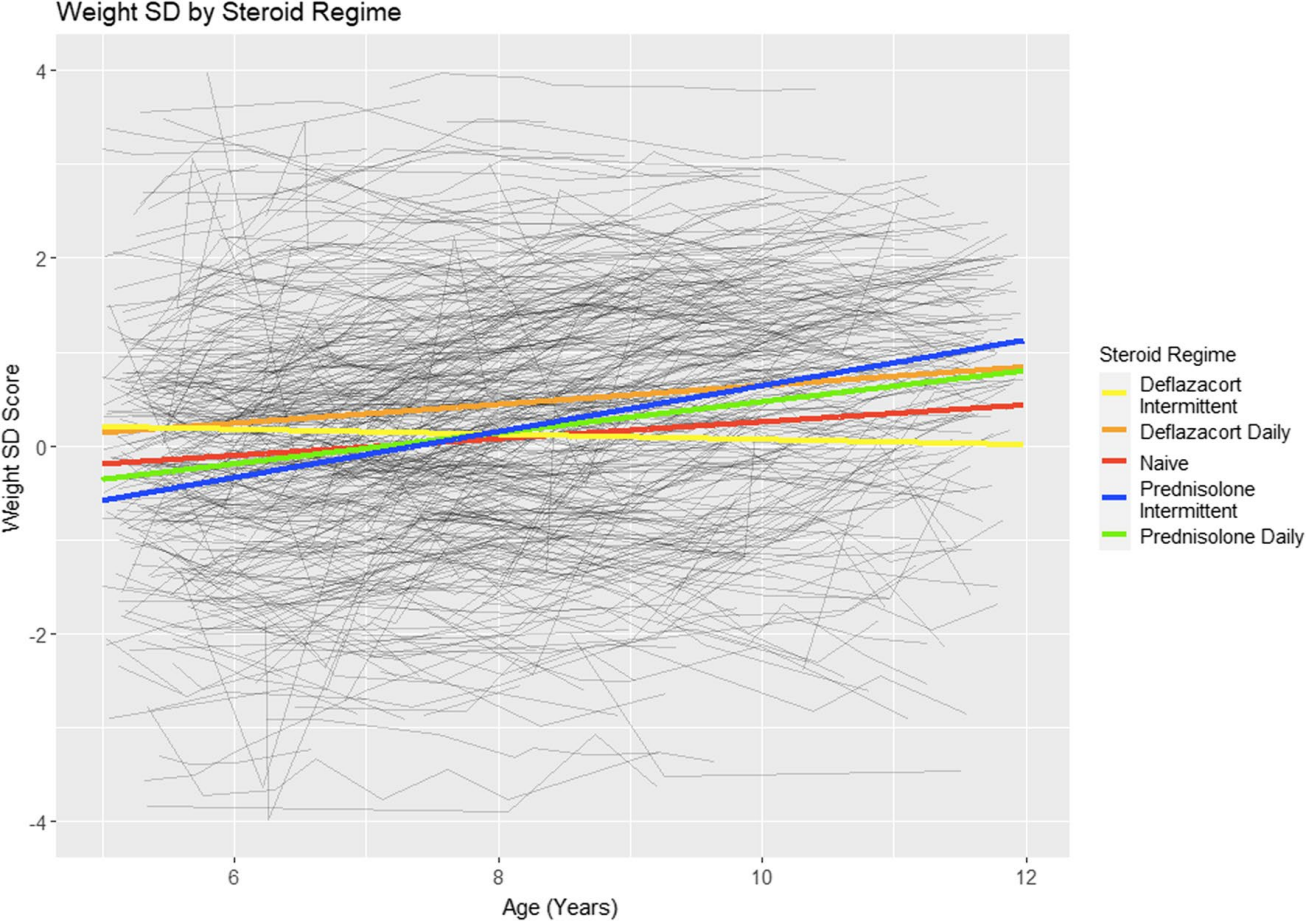
Mean weight SD trajectories by GC regime

There was a significant difference in weight at 5 years of age between the 5 majority GC treatment groups (“GC naïve”, “deflazacort daily”, “deflazacort intermittent”, “prednisolone daily” and “prednisolone intermittent”) (p < 0.01). The deflazacort subgroup had significantly higher weight at age 5 compared to the prednisolone subgroup (p < 0.01). However, the prednisolone subgroup showed a higher yearly weight gain compared to the deflazacort subgroup (p < 0.001). There was no significant difference between the regimes subgroups (intermittent or daily) in both weight at 5 years of age (p = 0.09) and yearly weight gain (p = 0.09). The average trajectories for the five GC groups are shown in Fig. 2. The estimated trajectories for all three growth measures are presented in Table 3. Earlier GC starting age was significantly associated with higher weight at age 5 but a smaller yearly increase in weight. For every year earlier a patient was started on GC they were 0.28 SD (95% CI 0.20, 0.37) heavier at age 5 but had a 0.03 SD (95% CI 0.01, 0.04) lower yearly weight gain.

**Table 3:** Parameter estimates for longitudinal models of height, weight and BMI SD by GC groups

	Height	Weight	BMI
	Est mean SD at age 5 years	Est mean SD at age 5 years	Est mean SD at age 5 years
Naive	− 0.84 (− 1.19, − 0.48)	− 0.2 (− 0.63,0.23)	0.64 (0.23,1.06)
Daily deflazacort (DD)	− 0.91 (− 1.13,− 0.68)	0.14 (− 0.14,0.42)	1.18 (0.92,1.44)
Intermittent deflazacort (ID)	− 0.69 (− 1.06, − 0.33)	0.2 (− 0.25,0.65)	0.92 (0.5,1.34)
Daily prednisolone (DP)	− 0.94 (− 1.12, − 0.77)	− 0.36 (− 0.57,− 0.15)	0.59 (0.39,0.79)
Intermittent prednisolone (IP)	− 1.21 (− 1.41, − 1.02)	− 0.59 (− 0.82,− 0.35)	0.42 (0.2,0.64)
All patients	− 0.96 (− 1.06, − 0.86)	− 0.27 (− 0.4,− 0.14)	0.68 (0.56,0.79)

	Est change in SD per year (95% CI)	Est change in SD per year (95% CI)	Est change in SD per year (95% CI)
Naive	0.03 (− 0.07, 0.13)	0.09 (− 0.05,0.23)	0.02 (− 0.11,0.16)
Daily deflazacort (DD)	− 0.25 (− 0.30, − 0.21)	0.1 (0.04,0.16)	0.2 (0.14, 0.26)
Intermittent deflazacort (ID)	− 0.06 (− 0.14, 0.02)	− 0.03 (− 0.13,0.07)	− 0.02 (− 0.12, 0.09)
Daily prednisolone (DP)	− 0.16 (− 0.19, − 0.12)	0.17 (0.12,0.21)	0.22 (0.18, 0.27)
Intermittent prednisolone (IP)	0.12 (0.08, 0.15)	0.25 (0.2,0.29)	0.17 (0.12, 0.22)
All patients	− 0.08 (− 0.1, − 0.06)	0.16 (0.13,0.19)	0.18 (0.15, 0.2)
Daily versus intermittent			
Difference in SDS at age 5 years (I-D)	− 0.16 (− 0.38,0.06) P = 0.16	− 0.24 (− 0.51,0.03) P = 0.09	**− 0.28 (− 0.54, − 0.03)** **P = 0.03**
Difference in annual change (I-D)	**0.27 (0.22, 0.31)** **P < 0.01**	0.05 (− 0.01,0.11) P = 0.09	**− 0.08 (− 0.14, − 0.02)** **P < 0.01**
Deflazacort versus prednisolone			
Difference in SDS at age 5 years (P-D)	− 0.2 (− 0.43,0.04) P = 0.10	**− 0.61 (− 0.89, − 0.32)** **P < 0.01**	**− 0.59 (− 0.86, − 0.32)** **P < 0.01**
Difference in annual change (P-D)	**0.16 (0.11, 0.22)** **P < 0.01**	**0.13 (0.07, 0.19)** **P < 0.01**	0.05 (− 0.01, 0.12) P = 0.07
Age at starting GCs			
Change in SDS at age 5 years per year later GCs initiated	**− 0.1 (− 0.17, − 0.02)** **P = 0.01**	**− 0.27 (− 0.36, − 0.18)** **P < 0.01**	**− 0.27 (− 0.36, − 0.18)** **P < 0.01**
Annual change in SDS per year later GCs initiated	0.01 (0, 0.03) P = 0.07	**0.03 (0.01, 0.04)** **P < 0.01**	**0.03 (0.01, 0.05)** **P = 0.01**

Bold signifies significant difference in subgroups (*p* < 0.05)

### Height

**Fig. 3:**
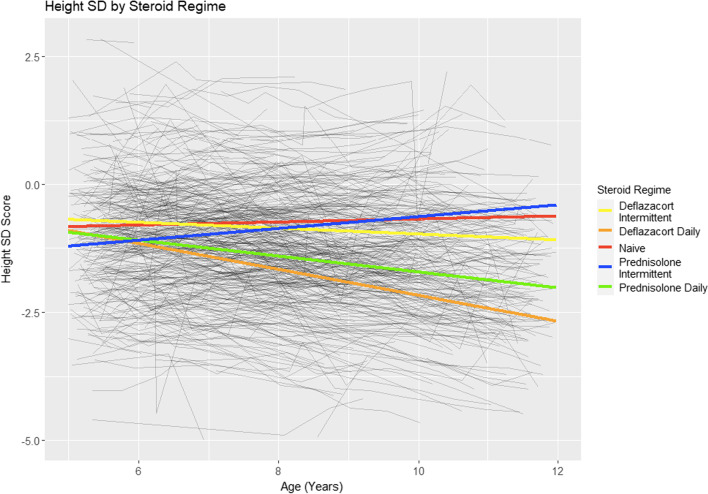
Mean height SD trajectories by GC regime

There was no significant difference in height at 5 years of age or mean annual change in height SD between the on GC and GC naïve subgroups. However, both the deflazacort daily and prednisolone daily subgroups showed significant yearly stunting of height compared to the GC naïve population (p < 0.01 and p < 0.01, respectively), and showed more stunting compared to their corresponding intermittent regimes. The daily deflazacort subgroup had the greatest stunting of height relative to the general paediatric population, losing − 0.25 (95% CI − 0.30, − 0.21) SD every year. The average height trajectories for the 5 GC groups are shown in Fig. 3. GC starting age was not significantly associated with a difference in height trajectory with time.

### BMI

**Fig. 4:**
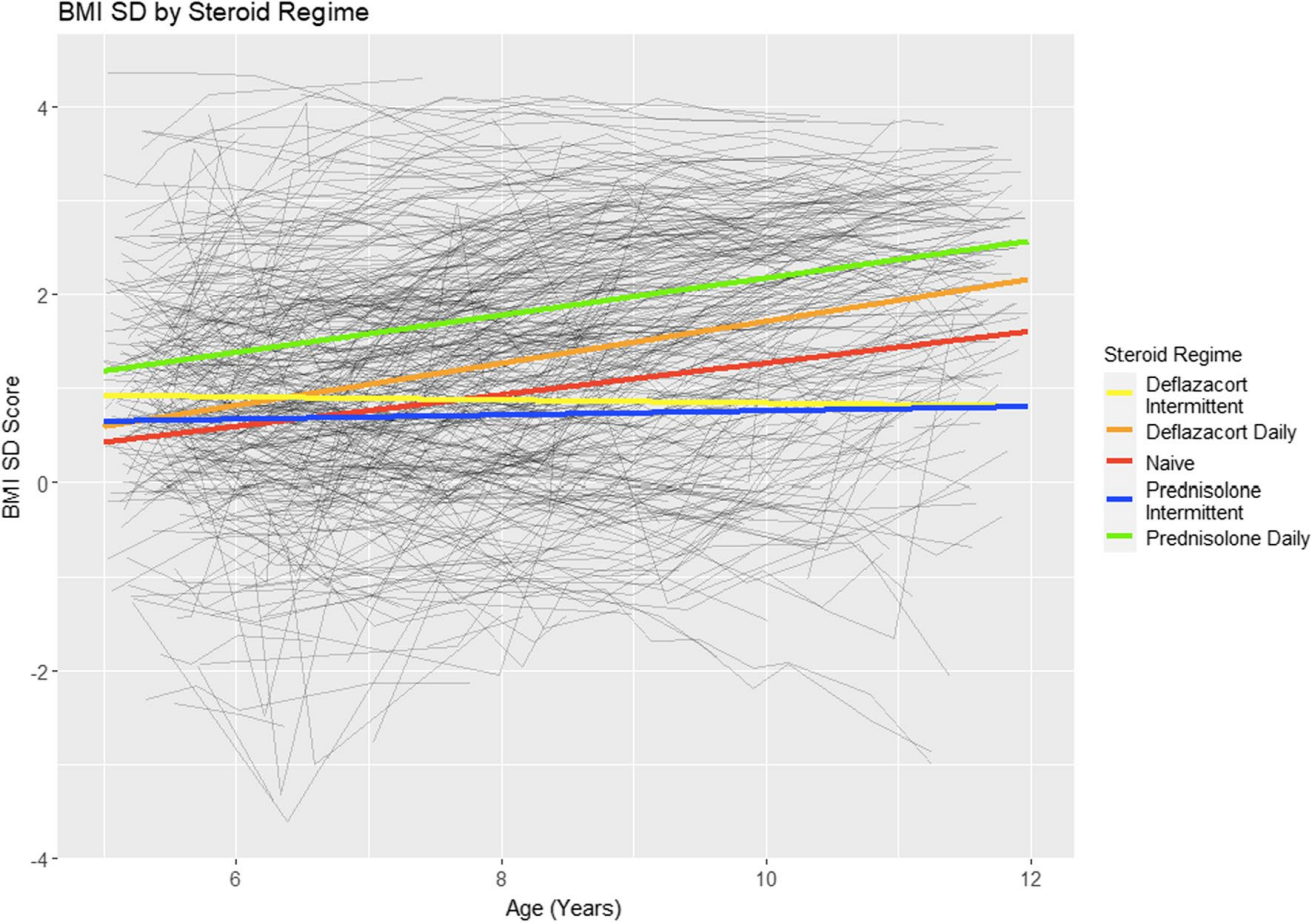
Mean BMI SD trajectories by GC regime

We found a significant difference in BMI at age 5 years between the GC regime groups (p < 0.01), with the prednisolone daily subgroup having the highest BMI at age 5. The deflazacort daily, prednisolone daily and prednisolone intermittent subgroups had on average a yearly BMI gain significantly greater than the GC naive group (0.18 (p = 0.02), 0.2 (p = 0.01) and 0.15 (p = 0.05), respectively). The average BMI trajectories for the 5 GC groups are shown in Fig. 4. Earlier GC starting age was significantly associated with higher BMI at age 5 but a smaller yearly increase in BMI. For every year later a patient was started on GC, they were observed to have a BMI score 0.27 SD (95% CI 0.18, 0.36) lower at age 5 but with a yearly BMI gain 0.03 SD (95% CI 0.01, 0.04) higher.

### Growth patterns according to DMD pathogenic variants

The majority of participants were in group 1 (Dp427 absent, Dp140/Dp71 present) or group 2 (Dp427/Dp140 absent, Dp71 present), and a minority of patients had a pathogenic variant relating to the expression of Dp71. Participants in group 3 (Dp427/Dp140/Dp71 absent) were significantly shorter (p < 0.01) than those in group 1 and group 2, by 0.82 (95% CI 1.28,0.36) and 0.77 (95% CI 0.3, 1.24) SD, respectively at all age points. There was no significant difference in height between those in group 1 and group 2 (p = 0.66). There was no significant difference in either weight or BMI at the age of 5 years or yearly growth between the 3 dystrophin isoforms groups. There were no differences in any growth parameter between the groups with DMD mutations amenable to skipping of exons 8, 44, 45, 51 or 53.

### Impact of growth patterns on loss of ambulation

For ambulant boys in each of the 6 age categories (5–6, 6–7, 7–8, 8–9, 9–10, 10–11), weight did not significantly affect the risk of loss of ambulation. For ambulant boys in the five youngest age categories, height did not significantly affect the risk of loss of ambulation. However, for ambulant boys between the age of 10 and 11, height significantly (p = 0.03) affected the risk of loss of ambulation. That is, for ambulant boys between the ages of 10 and 11 for every 0.25SD taller the patient was there was an associated 6% increase in the risk of losing ambulation during follow up. Analysis of older patients (such as the 11–12 age group) was limited by small sample numbers.

## Discussion

To our knowledge, this is the first longitudinal study investigating the growth patterns of a large cohort of DMD patients according to genotype, GC types and regimes. The NorthStar Database is one of the largest DMD databases, including all the Neuromuscular centres in the UK regularly monitoring DMD patients according to the SoC recommendations. It is worth noting that only a subset of the entire NorthStar population were included in the analysis due to missing data, and we cannot exclude that certain subpopulations may be underrepresented in the analysis, which could potentially induce bias. For example, in our cohort there were more participants in the prednisolone subgroup than in the deflazacort subgroup. Prednisolone has been more widely used in the UK in the last 20 years, while deflazacort has only started to be prescribed in clinic more recently. This is due to the increasing body of evidence on the effects of prolonged ambulation and reduced weight gain compared to prednisolone. There is also some imbalance in covariate group sample sizes, with certain covariate subgroups having a significantly smaller sample size, for example the deflazacort intermittent, Dp71- or the exon 8 skipping amenable group. Whilst small covariate group sizes may present issues in terms of overfitting, any potential bias induced by the small covariate group sample sizes in this study is likely negligible. Despite the limitation of this analysis being retrospective, the use of the data from the NorthStar Database provides valuable clinical information. As reported by previous studies, the distribution of the participants confirms that DMD patients tend to have higher weight [24] and lower height compared to the general paediatric population [25, 26]. This also affects BMI, which shows a greater spread across the age span, and tends to increase with age.

Looking at the growth pattern trajectories of DMD boys between the age of 5 and 12 years (the range most typically corresponding to the ambulatory disease phase), we found that those on deflazacort had significantly higher weight at age 5 years than those on prednisolone (p < 0.01). This may be related to the fact that the clinical decision to prescribe deflazacort rather than prednisolone might have been driven by the children’s characteristics. In the absence of a consensus on the optimal GC to prescribe, the clinical choice is often dictated by the desire to gain the maximum clinical and functional benefits while limiting long-term side effects like excessive weight gain and behavioural changes. Therefore, a plausible explanation for our findings is that the higher weight at 5 years in the deflazacort group might have been biased by the decision to start deflazacort in children who were already overweight. However, as previously reported [27, 28], the prednisolone subgroup showed a higher yearly weight gain than the deflazacort subgroup (p < 0.01). Unlike previous studies [29], we did not find a significant difference in the yearly weight gain rate between the daily or intermittent subgroups. This may, be due to the omission of GC dose in this analysis, as those on intermittent regimes typically have a higher mg/kg dose than those on daily [12]. In this observational study, we found that age at GC commencement was associated with different rates of weight gain. Every year earlier starting GC was associated with weight at age 5 0.28 SD (95% CI 0.20, 0.37) higher, but a yearly weight gain that was 0.03 SD lower (95% CI 0.01, 0.04).

Of note, GC initiation at earlier ages has become more common in recent years due to the increasing evidence in promoting functional benefits and prolonging ambulation [14, 18]. In the absence of long-term data from a randomised study, our findings should provide reassurance to clinicians that early commencement of GC treatment does not have more detrimental effect with regards to long-term weight gain.

Our data confirms that DMD children are on average shorter than the UK reference population at age 5 years, by 0.86 (95% CI 0.77, 0.96) SD. Height in DMD boys has been reported to be normal at birth, with a progressive slowdown in growth in the first years of life, but the underlying biological mechanism is still unknown [25]. In particular, skeletal disproportion has been noted [30]. Irrespective of the GC type, daily regimes showed the highest yearly stunting of height. As shown in previous studies [18, 27, 28, 31] participants on daily deflazacort had the greatest stunting of height, showing a loss of 0.25 (95% CI 0.21, 0.3) SD every year compared to the UK 90. However, unlike weight, GC start age was not significantly associated with yearly height change.

Boys with DMD are significantly shorter than boys with Becker muscular dystrophy [32]. Additionally, those with affected expression of Dp71 had significantly higher incidence of short stature when examined cross-sectionally at 6 years [32]. Our work supports and advances this conclusion, by demonstrating that boys with affected expression of Dp71 are, on average, 0.82 (95% CI 0.36, 1.28) SD shorter at all time points than those with the most proximal pathogenic variants, where only Dp427 expression was affected, and 0.77 (95% CI 0.30, 1.24) SD shorter than those with Dp427 and Dp140 expression affected. We found that there was no difference in association between weight and GCs across the three genotype groups. A possible explanation for the lower height trajectory observed in the DMD subpopulation where the ubiquitously expressed Dp71 dystrophin isoform was affected could be related to the involvement of Dp71 in cellular proliferation. Previous studies have documented a marked delay in cell growth in Dp71-knockdown cells [33]. Dp71 has been reported to modulate cell division and to regulate cell cycle through its contribution to the formation of the mitotic spindle and its involvement in multi-protein apparatuses of cytokinesis [33]. Another possible explanation could be related to the fact that Dp71 is the major brain product of the DMD gene and is expressed in ubiquitous tissues including the hypothalamo–neurohypophyseal axis [34] and may thus be implicated in endocrine regulation of growth. Further pathophysiology study will be necessary to demonstrate these hypotheses.

We found that there was no significant difference in the impact on yearly BMI gain between deflazacort and prednisolone; however, age at starting GC affected BMI with participants starting GC earlier having an average higher BMI SD at the age of 5 years than those starting later, but with a lower yearly gain.

The impact of growth on loss of ambulation is debated. Our study showed that increased weight was not associated with an earlier loss of ambulation. This is in line with previous studies showing that the beneficial effect of GC on muscle strength in DMD are also associated with an increase in muscle mass, which is in turn mediated by inhibition of muscle proteolysis rather than stimulation of muscle protein synthesis [35]. Similarly, there was no association between height and loss of ambulation under the age of 10 years. However, ambulant boys between the age of 10 and 11 years, were 6% more at risk of losing ambulation during follow up for every 0.25SD taller they were. These findings represent further elements to inform and counsel parents and patients about long term prognosis in DMD particularly in relation to GC treatment. The possible association between height and loss of ambulation has been previously explored but no definite conclusions have been made. A milder DMD phenotype in a patient with growth hormone deficiency was observed [36], and a randomised control trial giving the human growth hormone inhibitor Mazindol to monozygotic twins showed significant differences in motor function over the 1-year follow up [37]. However, a larger trial with 55 DMD boys failed to find significant differences between the Mazindol and placebo groups [38]. A limitation of our analysis is that we were not able to adjust for GC regimes or starting age, while previous studies have shown that daily GC regimes were associated with delayed loss of ambulation compared to intermittent regime [18].

Another possible limitation is that our findings might be partly related to the non-standardised method of measuring height that could have led to differences in detection between operators, as well as the possible presence of ankle contractures and/or the difficulties of patients to keep their heels on the ground during height measurements due to disease progression at older ages. Prospective, multicentre studies based on standardised, disease-specific protocols for collecting anthropometric measures are needed to overcome intra- and inter-operator differences in data collection [39].

In conclusion, our study found that participants on prednisolone longitudinally had a higher yearly weight gain compared to those on deflazacort, with no difference between intermittent versus daily regimes. Age at GC commencement plays a role with those starting at a later age showing higher rate of yearly weight gain. Daily deflazacort was associated with the greatest stunting of height compared to prednisolone and naïve participants, with daily regimes leading to higher stunting than the intermittent regimes. Finally, genotype and dystrophin isoform expression affect height trajectories as DMD boys with mutations affecting expression of Dp71 are shorter at all time points than those with more proximal pathogenic variants. These findings may provide further guidance to clinicians when counselling and discussing GCs commencement with patients and their carers and may represent a benchmark set of data to evaluate the effects of new generations of GC with differential mechanisms of action designed to limit mineralocorticoid-related side effects.

## Data Availability

The data referenced in this study are not publicly accessible, as it contains sensitive medical information. However, external parties can request data in aggregate form from the NorthStar network leads: Professor Muntoni, Dr Manzur and Dr Baranello.

## Abbreviations

BMI: Body Mass Index
DMD: Duchenne muscular dystrophy
FDA: Food and Drug Administration
GC: Glucocorticoids
SD score: Standardised Score
SoC: Standard of Care
